# Prehabilitation is effective in relieving pain after knee arthroplasty, but has little effect on length of stay and knee function: a meta-analysis of randomized controlled trials

**DOI:** 10.3389/fmed.2025.1457407

**Published:** 2025-04-28

**Authors:** Weishuai Zhang, Xuchao Lu, Nannan Yang, Xianyou Zhu, Haotian Hu

**Affiliations:** ^1^The Fifth Ward of Orthopedics Department, Kaifeng People’s Hospital, Henan, China; ^2^Department of Pain, Kaifeng People’s Hospital, Henan, China

**Keywords:** prehabilitation, knee arthroplasty, length of stay, knee flexion and extension, VAS score

## Abstract

**Purpose:**

The efficacy of preoperative rehabilitation (prehabilitation) for patients undergoing knee arthroplasty remains controversial. Prehabilitation is defined as the implementation of functional exercises, health education, and preemptive medication before surgery to improve postoperative outcomes, typically compared to conventional care protocols. Existing studies have reported inconsistent results regarding its benefits. This meta-analysis aims to evaluate the impact of prehabilitation on hospital length of stay, postoperative pain, and knee function in patients undergoing knee arthroplasty.

**Methods:**

PubMed, Cochrane, Embase, and Web of Science were searched from their establishment to 16 January 2024. An additional 19 articles were obtained by reading the relevant literature or by a reference search. All clinical randomized controlled trials (RCTs) related to the prehabilitation of total knee arthroplasty were included. All trials were analyzed by two independent reviewers, and the resulting data were analyzed using a random effects model and processed using Review Manager5.4 statistical software. The main outcome measures are as follows: visual analog scale(VAS), knee flexion and extension, and length of stay (LOS).

**Results:**

A total of 18 articles, encompassing 21 RCTs with 2,150 participants (1,167 in the prehabilitation group and 983 in the control group), were included. The analysis revealed that prehabilitation significantly reduced postoperative pain at 1, 3, and 6 months, as evidenced by lower VAS scores. Improvements in knee function were noted in terms of knee extension at 1 month and knee flexion at 3 months postoperatively. However, no significant difference was observed in the length of hospital stay.

**Conclusion:**

Prehabilitation before knee arthroplasty effectively alleviates postoperative pain and partially enhances knee function in the early postoperative period but does not significantly affect the length of hospital stay.

## Introduction

Osteoarthritis of the knee is the most prevalent degenerative disease, with an increasing incidence in recent years. In Western countries, its prevalence among adults exceeds 20%, making it a significant cause of work-related disability (1). The therapeutic strategy for knee osteoarthritis focuses on pain relief and joint function enhancement (2). Initial treatment modalities include medication, weight management, and functional exercise. In contrast, knee arthroplasty is the definitive treatment for end-stage osteoarthritis, offering substantial relief (3). However, knee arthroplasty is highly invasive and demands stringent health prerequisites for patients. Consequently, in 1940, scholars advocated utilizing the pre-surgical interval for early rehabilitative exercises to enhance postoperative outcomes and expedite recovery (4). This kind of preoperative rehabilitation is called pre-rehabilitation or prehabilitation (5). It includes functional exercise, health education, and preemptive medication. In contrast, traditional preoperative interventions often only include routine nursing measures such as education for a short time before surgery. In clinical studies, prehabilitation has produced inconsistent results regarding its impact on postoperative recovery.

While many studies report no significant benefits of prehabilitation on surgical outcomes, these conclusions are contested by some clinical experiences (6–8). In response, we performed the first comprehensive meta-analysis of full RCTs examining the clinical effects of prehabilitation in total knee arthroplasty patients. This study aims to evaluate the efficacy of prehabilitation in reducing postoperative pain, decreasing hospitalization duration, and improving joint function, thus offering evidence-based insights for clinical practice.

## Methods

This study strictly followed the PRISMA (Preferred Reporting Items for Systematic Reviews and Meta-Analyses) guidelines for systematic review and meta-analysis. The specific process included literature search, screening, data extraction, and quality assessment, and the complete checklist is shown in Table 1.

**Table 1:** Characteristics of included articles.

Reference	Country	No. of patient		Intervening measure	Outcome indicator	Withdrawals and dropouts		Jadad score
		EG	CG			EG	CG	4
(11)	Canada	65	66	Exercise and Education	LOS	14	10	7
(12)	Spain	22	22	High-intensity resistance training	VAS ROM	0	0	4
(13)	Taiwan	126	117	Home rehabilitation education	LOS VAS	0	0	4
(14)	Denmark	30	29	Progressive resistance training	VAS ROM	1	7	6
(15)	United Kingdom	60	61	Acupuncture	LOS	3	0	7
(15)	United Kingdom	60	61	Physiotherapy	LOS	10	0	7
(16)	Greece	10	10	Blood-Flow Restriction Training	ROM	0	0	4
(17)	Thailand	30	30	Quadriceps Exercise	VAS ROM	0	0	7
(18)	Italy	61	61	Home exercise program	LOS	0	0	5
(19)	United Kingdom	322	150	Preoperative patient education	LOS	0	0	3
(20)	Japan	14	15	Body weight resistance cycle ergometer exercise.	ROM	0	0	4
(21)	Spain	26	26	Balance training	ROM	0	0	4
(21)	Spain	25	26	Balance training	ROM	0	0	4
(22)	Iran	86	85	Celecoxib	VAS	4	3	5
(22)	Iran	87	85	Gabapentin	VAS	5	3	5
(23)	Italy	15	15	I-ONE therapy	VAS	0	0	4
(24)	Thailand	48	44	Quadriceps exercise, diet control	VAS	0	4	4
(25)	Saudi Arabia	25	25	Physical therapy	VAS	0	0	4
(26)	United Kingdom	13	12	Psychological intervention	VAS	0	0	7
(27)	Australia	21	20	Physiotherapy	LOS	0	0	7
(2)	Turkey	21	23	Education and home-based exercise	VAS	0	0	3

### Trial design

Preoperative waiting time provides a window to optimize and influence the patient’s muscle strength, function, and health-related quality of life, which are often considered predictors of correlation with postoperative outcomes, and this preoperative enhancement of relevant factors is called prehabilitation (9). We conducted a comprehensive search for all available clinical randomized controlled trials (RCTs) investigating the effects of prehabilitation on knee replacement outcomes, encompassing multimodal interventions from the inception of the databases until 16 January 2024. PICOS framework was used to analyze articles: population (patients undergoing total knee arthroplasty), intervention (pre-rehabilitation), control (usual care or no pre-rehabilitation), outcome (VAS score, knee range of motion, and length of hospital stay), and study type (randomized controlled trial, RCT). The interventions examined included functional exercises, physical therapy, acupuncture, health education, and medications. Our primary aim was to evaluate the impact of these prehabilitation measures on postoperative outcomes, specifically assessing visual analog scale (VAS) scores, knee flexion/extension range, and hospital stay duration at various postoperative intervals.

### Inclusion and exclusion criteria

The inclusion criteria were as follows: (1) Study type: Randomized Controlled Trials (RCTs) only, as these provide the highest level of evidence; (2) Population: Studies involving patients undergoing primary total knee arthroplasty (TKA) due to osteoarthritis or similar conditions; (3) Intervention: Prehabilitation programs, including but not limited to exercise regimes, strength training, or any structured preoperative physical activity; and (4) Comparators: Standard care or no prehabilitation intervention. The exclusion criteria were as follows: (1) Study design: Non-randomized studies, observational studies, case reports, reviews, and editorials; (2) Population: Studies involving patients with conditions other than osteoarthritis or those undergoing revision TKA or other types of knee surgeries; (3) ^Intervention: Studies not focusing on prehabilitation or combining prehabilitation with other major interventions that do not isolate the effect of prehabilitation. All articles were reviewed jointly by two investigators to decide on inclusion, with a third investigator assisting in the decision if the two investigators did not agree on inclusion.

### Information sources

Database searches were performed independently by two researchers in PubMed, Embase, Cochrane Library, and Web of Science from their inception to 16 January 2024. Additional literature was identified through cross-referencing and review of relevant citations. The search terms employed included “Arthroplasty,” “Replacement,” “Knee,” and “Preoperative Exercise”.

The exact search process is as follows: (Arthroplasty, Replacement, Knee) OR (Arthroplasties, Replacement, Knee) OR (Arthroplasties, Replacement, Knee) OR (Knee Replacement Arthroplasties) OR (Knee Replacement Arthroplasty) OR (Replacement Arthroplasties, Knee) OR (Replacement Arthroplasties, Knee) OR (Replacement Arthroplasties, Knee) OR (Total Knee Arthroplasty) OR (Total Knee Arthroplasty) OR (Total Knee Replacement) OR (Knee Replacement, Total) OR (Knee Arthroplasty) OR (Arthroplasty, Knee) OR (Arthroplasties, Knee Replacement) OR (Arthroplasties, Knee Replacement) OR (Arthroplasty, Replacement, Partial Knee) OR (Unicompartmental Knee Arthroplasty) OR (Unicompartmental Knee Arthroplasty) OR (Knee Arthroplasty, Unicompartmental) OR (Unicondylar Knee Arthroplasty) OR (Arthroplasty, Unicondylar Knee) OR (Knee Arthroplasty, Unicondylar) OR (Knee Arthroplasty, Unicondylar) OR (Knee Arthroplasty, Unicondylar) OR (Knee Arthroplasty, Partial) OR (Unicondylar Knee Replacement) OR (Knee Replacement, Unicondylar) OR (Partial Knee Replacement) OR (Knee Replacement, Partial) OR (Unicompartmental Knee Replacement) OR (Knee Replacement) And (Preoperative Exercise) OR (Exercise, Preoperative) OR (Preoperative Exercises) OR (Pre-operative Conditioning) OR (Conditioning, Pre-operative) OR (Pre operative Conditioning) OR (Pre-operative Conditionings) OR (Pre-operative Rehabilitation) OR (Pre operative Rehabilitation) OR (Pre-operative Rehabilitations) OR (Rehabilitation, Pre-operative) OR (Preoperative Rehabilitation) OR (Preoperative Rehabilitations) OR (Rehabilitation, Preoperative) OR (Preoperative Conditioning) OR (Conditioning, Preoperative) OR (Preoperative Conditionings) OR (Pre-operative Exercise) OR (Exercise, Pre-operative) OR (Pre operative Exercise) OR (Pre-operative Exercises) OR (Prehabilitation).

### Data extraction

Data collection was independently carried out by two investigators using a standardized form, with a third investigator verifying the collected data for accuracy. The primary outcome of this study was the postoperative visual analog scale (VAS) scores. Secondary outcomes included the knee flexion and extension angles (degrees) and the length of hospital stay (days). Studies employing other scoring criteria were excluded from this analysis.

### Assessment of study quality

To assess the quality of the publications of the included studies, the modified Jadad scoring system was used, consisting of four main dimensions: generation of random sequences, concealment of randomization, blinding, and dropout and lost to follow-up, with the modified Jadad scale ranging from 0 to 7. A study was considered high quality when the score was between 4 and 7; when the score was between 1 and 3, the study was considered low quality.

### Risk of bias

The risk of bias in each included study was assessed independently by two investigators using the Cochrane Risk of Bias Tool (10). The outcomes were categorized as high risk, uncertain risk, or low risk. These assessments were then subjected to further review by a third investigator to ensure accuracy and consistency.

### Risk assessment of non-effectiveness of interventions

The methodological quality and risk of ineffectiveness of the included studies were comprehensively evaluated using the i-CONTENT tool, which assessed six dimensions: study design, sample size, intervention description, outcome measurement, data completeness, and risk of bias. The total score ranged from 0 to 100 points, with risk levels categorized as low (≥85 points), moderate (75–84 points), and high (≤74 points).

### Statistical analysis

Statistical analysis was performed using Review Manager (Version 5.4). Given the diversity of interventions and demographic data sources, a random effects model was applied. Continuous variables were expressed as weighted mean difference (WMD) or standardized mean difference (SMD), with 95% confidence intervals (95% CIs) calculated accordingly. The standard error (SE) was converted to standard deviation (SD) using the Evidence-Based Medicine Data Extraction Excel Universal Conversion Template 2.0, applicable when SE was reported instead of SD. The meta-analysis focused on VAS scores at 1, 3, and 6 months post-surgery, knee flexion and extension at 1 and 3 months post-surgery, and the length of hospital stay. Studies with inconsistent scoring timelines were excluded from the analysis. Subgroup analysis was planned for different prehabilitation interventions (e.g., exercise vs. medication vs. psychology); however, there were insufficient studies for each individual intervention for statistical validity.

## Results

### Study characteristics

**Figure 1:**
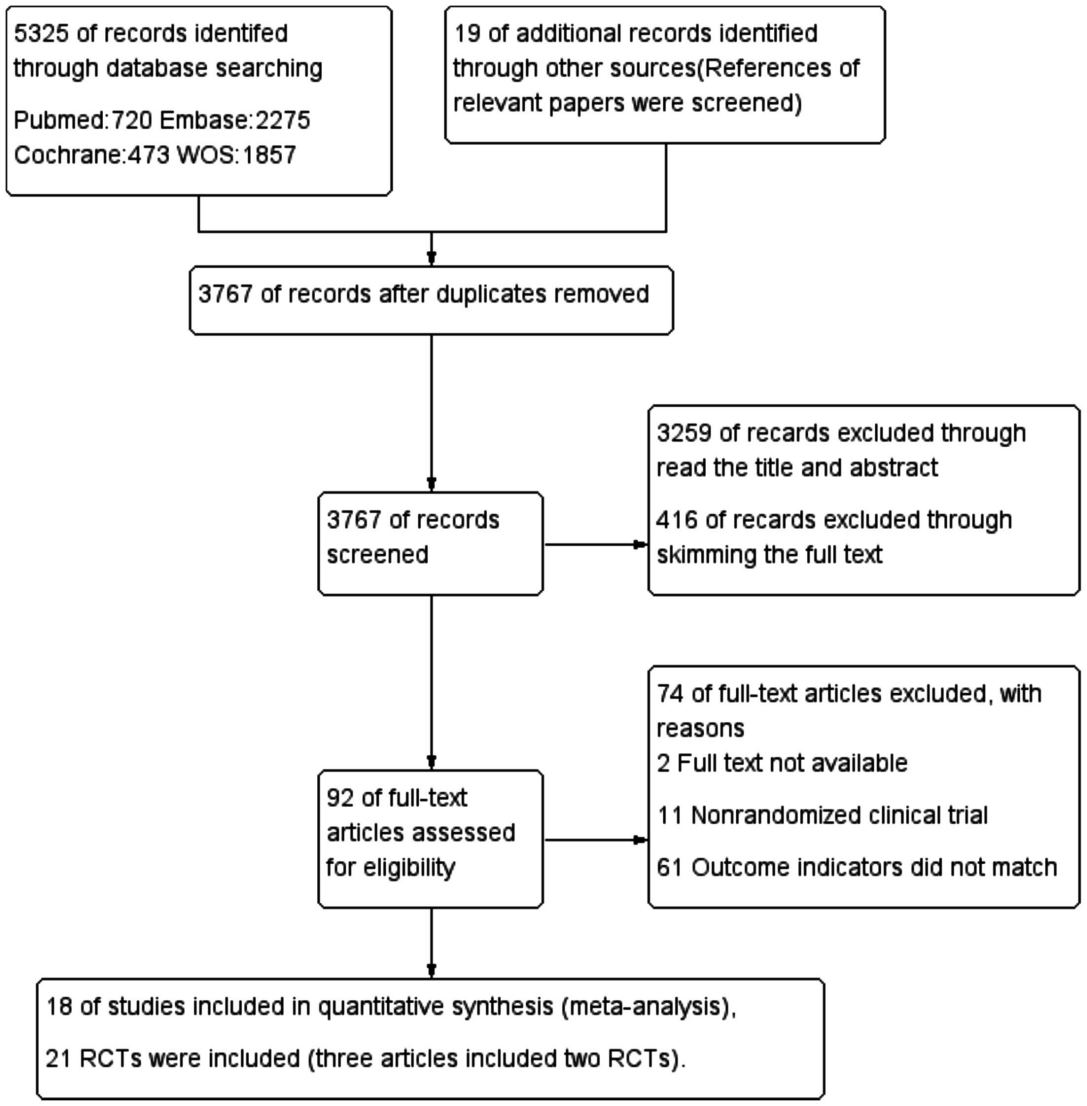
Study flow diagram.

A total of 5,325 articles were identified through database searches, with an additional 19 articles found through related literature and their references (Figure 1). After removing duplicates, 3,676 articles remained. Screening of titles and abstracts led to the exclusion of 3,259 articles; 416 records were excluded through skimming the full text, leaving 92 full texts for detailed review. Of these, 74 were excluded, resulting in 18 articles being ultimately included in the final meta-analysis. These comprised 21 controlled trials (2, 11–27), involving 2,150 patients (Prehabilitation: 1,167; Unprehabilitation: 983). Seven trials reported variable numbers of lost participants (Experimental Group: 29, Control Group: 20), and one article (14) did not specify the reasons for attrition. The characteristics of the selected articles are presented in Table 1.

Six articles (33%) assessed the length of stay (LOS); four articles (22%) evaluated VAS scores at 1 month post-surgery; eight articles (44%) analyzed VAS scores at 3 months post-surgery; five articles (28%) investigated VAS scores at 6 months post-surgery; five articles (28%) examined knee extension and flexion at 1 month post-surgery; and another five articles (28%) reviewed knee extension and flexion at 3 months post-surgery.

### Type of interventions

Eleven trials incorporated various functional exercises. These included muscle resistance exercises in nine trials (11–14, 17, 20, 21, 24, 27), joint flexion and extension in seven trials (2, 11–14, 16, 20), stair ambulation in six trials (11, 13, 18, 21, 27, 28), and balance exercises in four trials (11, 18, 21, 27). Four trials (2, 13, 19, 26) integrated preoperative education or a combination of education and exercise, which could be conducted at home or in the hospital. Medications such as celecoxib and gabapentin, along with acupuncture, were utilized in some studies (15, 16, 22). One trial (16) implemented Blood-Flow Restriction Training, while another (25) described a physical exercise regimen but did not detail the specific exercises involved.

### Risk of bias

**Figure 2:**
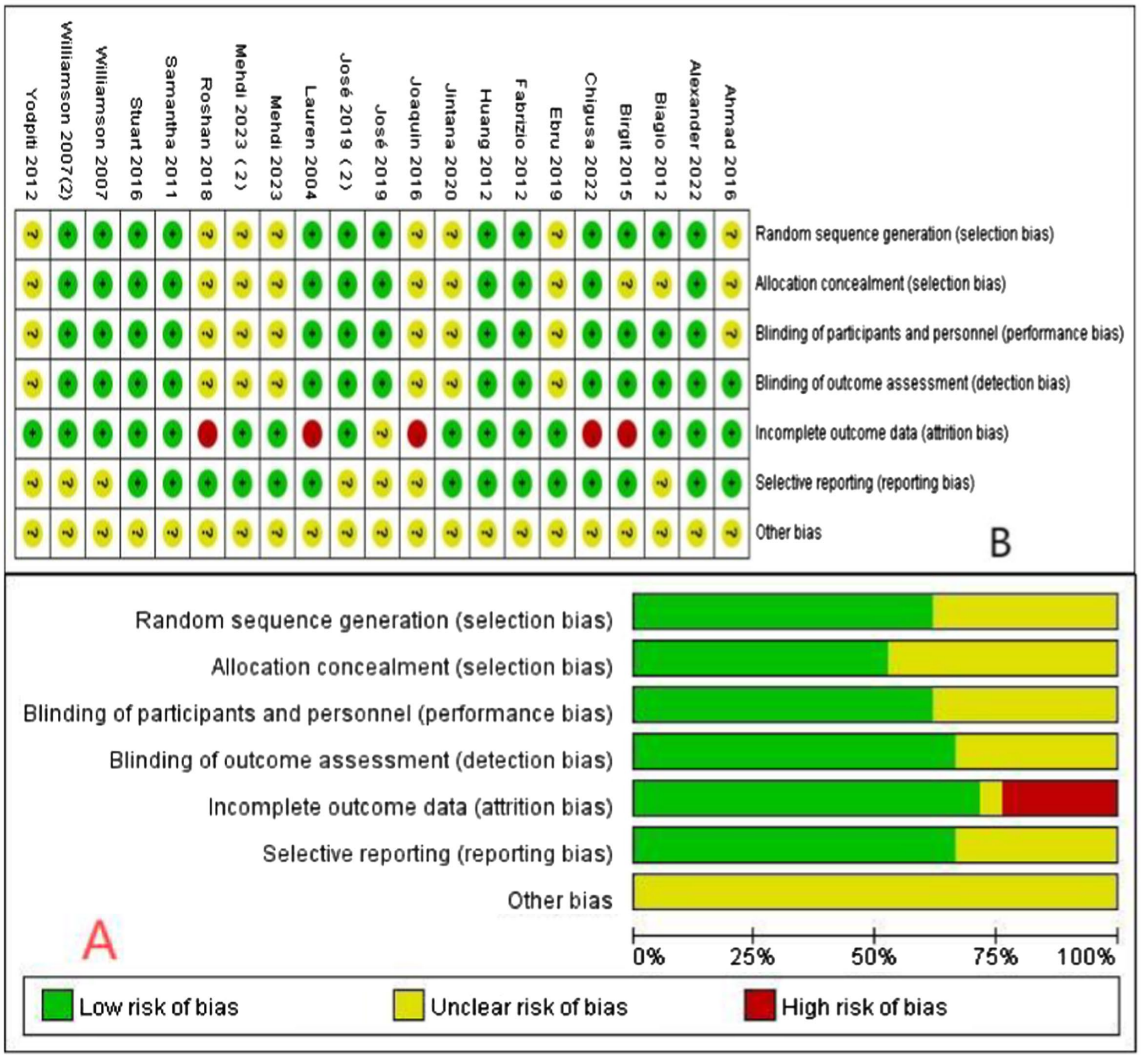
Assessment of the risk of bias in the included articles. **(A)** Risk of bias graph; **(B)** Risk of bias summary (“+”: low risk of bias; “?”: unclear risk of bias; “-”: high risk of bias).

The risk of bias assessment for the included articles is illustrated in Figure 2. In this systematic evaluation, we assessed the method of random sequence generation, patient informedness, and use of blinding in the included studies. Although some of the studies used reliable methods in randomized sequence generation, most of them lacked detailed descriptions of blinding in key aspects, which may lead to different degrees of selection bias and measurement bias. These bias factors need to be fully considered when interpreting study results. The specific risk of bias was analyzed as follows: a total of five articles used opaque envelopes to generate random sequences and four articles used computer-generated random sequences. However, nine articles did not specify the method of random sequence generation. This suggests that some studies may have been at risk of selection bias in the randomization process. In addition, patients in all studies were informed of the surgical method, suggesting that these studies failed to effectively mitigate selection bias during intervention delivery. In terms of the use of blinding, nine articles did not detail the blinding method used to mitigate selection bias and six articles did not adequately describe the blinding method used for outcome assessment. This further increases the risk of potential bias in the outcome assessment process.

To determine the presence of publication bias in the included studies, we plotted funnel plots for each of the included studies for each observational metric to check for the presence of publication bias; symmetrical funnel plots indicate no significant publication bias, whereas asymmetrical funnel plots indicate the possible presence of bias, and after interpreting the results of all the funnel plots, there was a more pronounced publication bias in all the articles except for the article that had a VAS score at 1 month postoperatively as the observational metric. Except for the articles with VAS score at 1 month after surgery as an observational indicator, there was no significant publication bias in any of the included studies for the other observational indicators.

Results of the I-CONTENT tool assessment demonstrated the risk of non-effectiveness of the included studies’ interventions. Among the 20 studies, total scores ranged from 76 to 95 points, with a mean score of 83.8. Low-risk studies accounted for 45% (9 studies), with a mean score of 88.3 and a maximum score of 95. Moderate-risk studies comprised 45% (9 studies), with a mean score of 80.3, while high-risk studies represented 10% (2 studies), with a mean score of 76.5 and a minimum score of 76. Although most studies (16 studies) provided clear descriptions of interventions, four studies lacked critical information, potentially compromising clinical reproducibility. Additionally, subjective measurement tools (e.g., patient self-assessment) in eight studies were not fully validated, which may reduce the objectivity of outcomes. This evaluation indicates that nearly half of the interventions (45%) demonstrated high-quality evidence, while an equivalent proportion required cautious interpretation within clinical contexts. Future research should prioritize expanding sample sizes, standardizing intervention descriptions, and enhancing bias control to improve reliability, and the complete checklist is shown in Table 2.

**Table 2:** Risk assessment form for i-CONTENT interventions.

Reference	Intervention measure	Study design (0–20)	Sample size (0–15)	Intervention description (0–20)	Outcome measurement (0–15)	Data completeness (0–15)	Risk of bias (0–15)	i-CONTENT total score (0–100)	Risk of ineffectiveness
(11)	Exercise and Education	15	12	18	12	15	14	86	Low
(12)	High-intensity Resistance	20	11	17	13	13	11	85	Low
(13)	Home Rehabilitation Education	15	15	16	11	14	11	82	Moderate
(14)	Progressive Resistance	20	11	17	14	12	13	87	Low
(15)	Acupuncture	20	15	15	13	14	14	91	Low
(15)	Physiotherapy	20	15	15	13	14	14	91	Low
(16)	Blood-Flow Restriction	15	10	15	14	13	13	80	Moderate
(17)	Quadriceps Exercise	20	10	18	13	14	14	89	Low
(18)	Home Exercise Program	16	12	16	12	12	14	82	Moderate
(19)	Pre-operative Education	15	15	15	12	14	14	85	Low
(20)	Body Weight Resistance	16	9	16	13	12	10	76	Moderate
(21)	Balance Training	15	11	15	12	14	11	78	Moderate
(21)	Balance Training	15	11	15	12	14	11	78	Moderate
(22)	Celecoxib	18	13	16	14	14	13	88	Low
(22)	Gabapentin	18	13	16	14	14	13	88	Low
(23)	I-ONE Therapy	16	10	14	12	13	12	77	Moderate
(24)	Quadriceps Exercise, Diet	15	12	15	13	14	12	81	Moderate
(25)	Physical Therapy	15	10	15	13	14	12	79	Moderate
(26)	Psychological Intervention	20	10	17	13	14	14	88	Low
(27)	Physiotherapy	20	10	18	14	14	14	90	Low
(2)	Education and Home Exercise	15	10	15	13	13	11	77	Moderate

### Association of prehabilitation with VAS score

**Figure 3:**
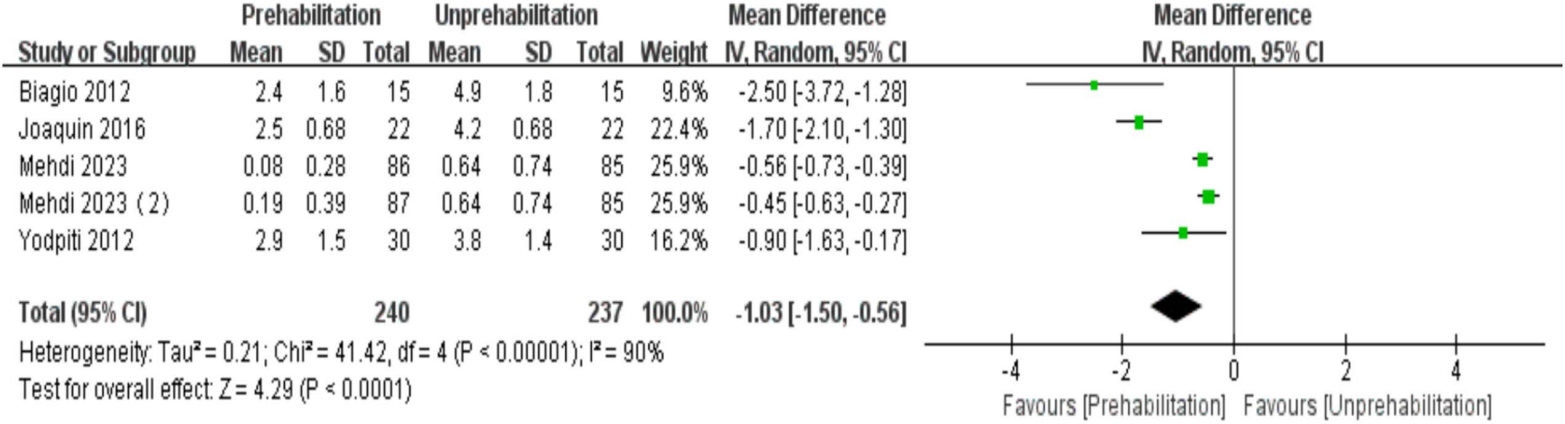
Forest plot of VAS score at 1 month after surgery.

One month post-surgery, the VAS score in the prehabilitation group was significantly lower than in the unprehabilitation group, with a notable statistical difference observed [5 trials, *n* = 477 (12, 17, 22, 23); mean difference: −1.03, (95% CI, −1.50 to-0.56), *p* < 0.0001; Figure 3].

**Figure 4:**
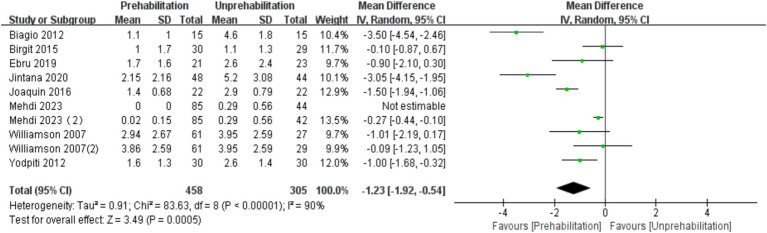
Forest plot of VAS score at 3rd month after surgery.

Three months post-surgery, the VAS score for the prehabilitation group was significantly lower than that of the unprehabilitation group, with a substantial statistical difference noted between the two groups [10 trials, *n* = 763 (12, 14, 15, 17, 22–24, 26); mean difference: −1.23, (95% CI, −1.92 to-0.54), *p* = 0.0005; Figure 4].

**Figure 5:**
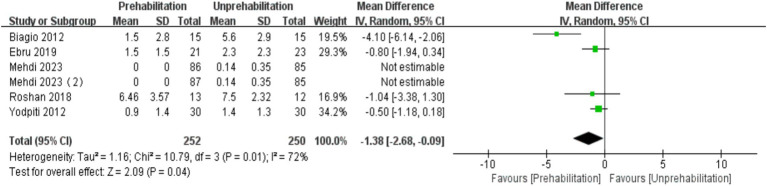
Forest plot of VAS score at 6th month after surgery.

Six months post-surgery, the VAS score for the prehabilitation group was significantly lower than that of the unprehabilitation group, with a marked statistical difference between the groups [6 trials, *n* = 502 (2, 12, 17, 22, 29); mean difference: −1.38, (95% CI, −2.68 to-0.09), *p* = 0.0004; Figure 5].

### Association of prehabilitation with LOS

**Figure 6:**
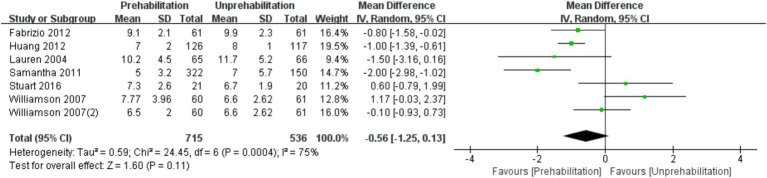
Forest plot of LOS.

Prehabilitation had no significant effect on the LOS [7 trials, *n* = 1,251 (11, 13, 15, 18, 19, 27), mean difference:-0.56, (95% CI, −1.25 to-0.13), *p* = 0.11; (Figure 6)].

### Association of prehabilitation with knee flexion and extension

**Figure 7:**
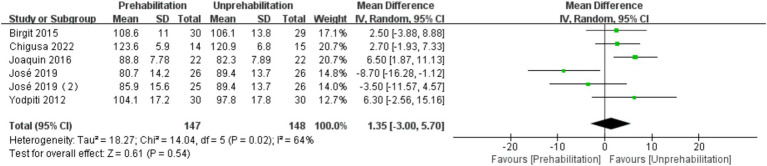
Forest plot of knee flexion at 1 month after surgery.

**Figure 8:**
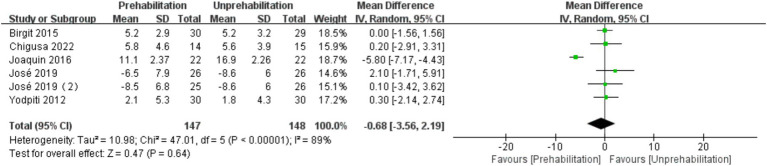
Forest plot of knee extension at 1 month after surgery.

**Figure 9:**
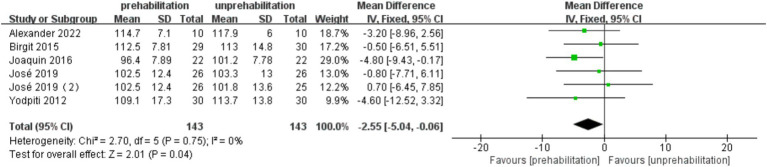
Forest plot of knee flexion at 3 months after surgery.

**Figure 10:**
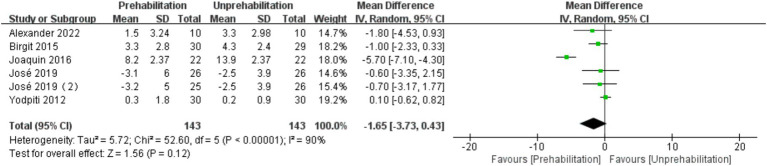
Forest plot of knee extension at 3 months after surgery.

Pre-rehabilitation had no significant impact on knee flexion [6 trials, *n* = 295 (12, 14, 17, 20, 21); mean difference: 1.35, (95% CI, −3.00 to 5.70) *p* = 0.12; see Figure 7] and extension [6 trials, *n* = 295 (12, 14, 17, 20, 21); mean difference: −0.68, (95% CI, −3.56 to 2.19), *p* = 0.54; see Figure 8] at 1 month post-surgery, nor on knee extension [6 trials, *n* = 286 (12, 14, 16, 17, 21); mean difference: −1.65, (95% CI, −3.73 to 0.43), *p* = 0.54; Figure 9] at 3 months post-surgery. However, it improved knee flexion function at 3 months post-surgery [6 trials, *n* = 286 (12, 14, 16, 17, 21); mean difference: 2.55, (95% CI, 0.06 to 5.04), *p* = 0.04; see Figure 10].

## Discussion

This meta-analysis represents the first systematic review of randomized controlled trials assessing prehabilitation in knee replacement surgery. It provides important evidence that preoperative rehabilitation is effective in relieving knee pain in patients up to 6 months postoperatively and improves knee flexion function at 3 months postoperatively. Nonetheless, the study encounters several limitations. Primarily, it included only clinical RCTs with predefined observation time points, resulting in a limited number of trials for each outcome and complicating the statistical analysis of additional indicators, thus potentially diminishing its clinical applicability. Moreover, the extensive time span covered by the study introduced the possibility of confounding and selection biases, particularly in earlier trials with design and operational irregularities. The preoperative exercise and rehabilitation programs in most studies were standardized and not patient-specific, which may not suit every individual’s needs, possibly explaining the lack of prehabilitation benefits for some patients. The diverse methodologies and interventions (including trial design, type of intervention, observation period, and follow-up duration) across the included trials contributed to significant heterogeneity. Furthermore, the inclusion of trials from 13 different countries and regions introduced variability in patient populations, geographic and cultural factors, and economic conditions, all of which could adversely affect the reliability of the results and increase heterogeneity.

Knee osteoarthritis is a leading cause of disability worldwide (28). While knee replacement surgery effectively reduces disability risk, the postoperative recovery process is lengthy and painful. The primary challenge for clinicians is optimizing patients’ physical and mental states pre-surgery to enhance compliance and ensure surgery is performed under optimal conditions (29). Evidence supporting prehabilitation for post-surgical recovery, especially after knee replacement, remains limited (30). Numerous studies indicate that pre-rehabilitation can significantly improve the range of motion (ROM) of the knee joint both before and after surgery (31, 32). However, a meta-analysis by Granicher et al. of 16 clinical trials (968 patients) suggested that prehabilitation improves knee function before and within 1 year after total knee replacement, particularly within the first 3 months post-surgery. Still, they reported the evidence level as low to very low (6). They posited that prehabilitation’s benefits for total knee replacement patients are primarily short-term and diminishing over time, concluding that it has no significant long-term effects on knee function post-TKA. However, Granicher’s study included more literature but with a lower level of evidence, and its interventions were mainly exercises to enhance knee function (mobility, resistance, sensory-motor, or endurance training), which differed considerably from the interventions in the literature included in our study (body weight, muscular strength, and balance). This was considered to be the main reason why the results of the analyses were not consistent with ours.

Contrary to these findings, our meta-analysis revealed that prehabilitation improved knee flexion function at 3 months post-surgery but had no significant short-term effect on knee flexion and extension functions, challenging the conclusions of previous authors. Current research on the impact of prehabilitation on hospital stay length after knee replacement is inconclusive. Still, the majority of studies suggest that pre-rehabilitation can reduce hospitalization time and associated costs (13, 15, 18, 19, 33). This reduction may be attributed to alleviated postoperative pain and enhanced muscle strength recovery. Our analysis determined that prehabilitation might shorten hospital stays, albeit not to a statistically significant degree. Regarding postoperative pain relief, prehabilitation’s effectiveness is relatively well-established (34), consistent with our study’s results. Nevertheless, some researchers contend that prehabilitation does not significantly impact pain relief after knee replacement (35). Our findings indicate that prehabilitation effectively mitigates postoperative pain in knee replacement patients, with the most pronounced effects within the first 6 months post-surgery and a gradual decrease over time.

## Conclusion

The results of meta-analysis showed that pre-rehabilitation significantly reduced postoperative pain, and VAS scores were significantly lower at each observation time point after surgery. VAS scores in the rehabilitation group were significantly lower than those in the non-rehabilitation group, and the difference was statistically significant: 1 month after surgery [mean difference: -1.03, 95% CI, −1.50 -- 0.56], *p* < 0.0001; 3 months after surgery (mean difference: -1.23,95% CI, −1.92 to-0.54, *p* = 0.0005); and 6 months after surgery (mean difference: -1.38, 95% CI, −2.68 to-0.09, *p* = 0.0004). The knee flexion function was improved at 3 months after operation (mean difference: 2.55, 95% CI, 0.06–5.04, *p* = 0.04). However, there was no significant effect on the length of hospital stay [mean difference: −0.56, (95% CI, −1.25 to-0.13), *p* = 0.11]. This is the first meta-analysis to comprehensively evaluate the impact of multimodal preadaptation, such as functional exercise, health education, and pharmacological interventions, on outcomes after total knee arthroplasty. A total of 21 large-sample randomized controlled trials (2,150 patients) were included, and the random effects model was used to reduce heterogeneity and improve statistical reliability. However, the intervention protocol of this study did not maintain uniform standards, the included studies span a wide range of time and regions, and some key indicators, such as quadriceps muscle strength, were not included in the final analysis, which may affect the reliability of the results. Therefore, due to the heterogeneity of interventions and methodological limitations, the results of this study should be interpreted with caution. Future studies should further standardize pre-rehabilitation protocols, extend follow-up periods, and incorporate patient-specific rehabilitation strategies to strengthen the evidence base. Although the effect of prehabilitation on length of stay remains statistically uncertain due to methodological heterogeneity and the limitation of a small sample, the consistent directional trend supports its exploratory clinical value. Future trials should prioritize standardized multimodal interventions, target high-risk subgroups, and stratify outcomes according to medical context to elucidate benefit.
